# Essential role of the iron-sulfur cluster binding domain of the primase regulatory subunit Pri2 in DNA replication initiation

**DOI:** 10.1007/s13238-015-0134-8

**Published:** 2015-02-04

**Authors:** Lili Liu, Mingxia Huang

**Affiliations:** 1 Department of Pharmacology and Chemical Biology, University of Pittsburgh School of Medicine, Pittsburgh, PA 15213 USA; 2 Department of Biochemistry and Molecular Genetics, University of Colorado School of Medicine, Aurora, CO 80045 USA

**Keywords:** primase, Pri2, iron-sulfur cluster, replication initiation

## Abstract

DNA primase catalyzes *de novo* synthesis of a short RNA primer that is further extended by replicative DNA polymerases during initiation of DNA replication. The eukaryotic primase is a heterodimeric enzyme comprising a catalytic subunit Pri1 and a regulatory subunit Pri2. Pri2 is responsible for facilitating optimal RNA primer synthesis by Pri1 and mediating interaction between Pri1 and DNA polymerase α for transition from RNA synthesis to DNA elongation. All eukaryotic Pri2 proteins contain a conserved C-terminal iron-sulfur (Fe-S) cluster-binding domain that is critical for primase catalytic activity *in vitro*. Here we show that mutations at conserved cysteine ligands for the Pri2 Fe-S cluster markedly decrease the protein stability, thereby causing S phase arrest at the restrictive temperature. Furthermore, Pri2 cysteine mutants are defective in loading of the entire DNA pol α-primase complex onto early replication origins resulting in defective initiation. Importantly, assembly of the Fe-S cluster in Pri2 is impaired not only by mutations at the conserved cysteine ligands but also by increased oxidative stress in the *sod1Δ* mutant lacking the Cu/Zn superoxide dismutase. Together these findings highlight the critical role of Pri2’s Fe-S cluster domain in replication initiation *in vivo* and suggest a molecular basis for how DNA replication can be influenced by changes in cellular redox state.

**Electronic supplementary material:**

The online version of this article (doi:10.1007/s13238-015-0134-8) contains supplementary material, which is available to authorized users.

## Introduction

A unique challenge in DNA replication initiation is the inability of DNA polymerases to synthesize new DNA strands *de novo* (Hubscher et al., 2002; Wang, 1996). The problem is overcome by DNA primase, a specialized DNA-dependent RNA polymerase that synthesizes a short RNA primer for DNA polymerase to extend new strand synthesis (Arezi et al., 1999; Arezi and Kuchta, 2000; Frick and Richardson, 2001; Zerbe and Kuchta, 2002). The eukaryotic DNA primase is part of the DNA polymerase α-primase (pol-prim) complex that is composed of four conserved subunits, referred to as Pol1, Pol12, Pri1 and Pri2 in the budding yeast *Saccharomyces cerevisiae* (Burgers, 1998; Foiani et al., 1997; Hubscher et al., 2002; Kawasaki and Sugino, 2001; Wang, 1996). The structural and catalytic properties of all four pol-prim subunits are highly conserved among eukaryotes; all four subunits are essential for viability in yeast cells (Budd and Campbell, 1987; Foiani et al., 1994; Foiani et al., 1989; Francesconi et al., 1991; Lucchini et al., 1987). The catalytic primase subunit Pri1 and its regulatory subunit Pri2 form the heterodimeric DNA primase. Pol1 is the catalytic subunit of DNA polymerase α that is tightly bound to its regulatory subunit Pol12, also known as the B subunit.

Replication of a eukaryotic genome is initiated from multiple specific sites along the chromosomes known as replication origins during the S phase of the mitotic cell cycle. The pol-prim complex associates with replication forks in S phase to initiate nucleic acid synthesis of the leading strand as well as each Okazaki fragment of the lagging strand (Muzi-Falconi et al., 2003). Primase synthesizes a 7–12 nt RNA primer (Arezi and Kuchta, 2000; Frick and Richardson, 2001) that is transferred to DNA Pol α for limited extension to ~30 nt. The resulting RNA-DNA hybrid primer is subsequently handed over to Pol ε and Pol δ on the leading and lagging strand, respectively, for processive DNA replication (Arezi et al., 1999; Hubscher et al., 2002; Zerbe and Kuchta, 2002).

The transition from primase-catalyzed RNA synthesis to Pol α-catalyzed DNA synthesis is mediated by Pri2 that bridges Pri1 and Pol α within the pol-prim complex (Arezi et al., 1999). Electron microscopy studies and computational modeling of the pol-prim complex reveal a dumbbell-shaped architecture with flexible association between the DNA polymerase and primase lobes (Nunez-Ramirez et al., 2011). Degradation of a Pri2 temperature-sensitive mutant protein at the restrictive temperature abolishes co-immunoprecipitation of Pol1-Pri1, whereas Pol1-Pri2 interaction remains intact when Pri1 is degraded (Longhese et al., 1993). Thus, the association between primase and Pol α appears to be directly mediated by Pri2, in keeping with co-immunoprecipitation studies of human pol-prim subunits that were ectopically expressed in insect cells (Copeland and Wang, 1993).

Pri2 also plays essential roles in stabilization of Pri1 as well as in the initiation, elongation and length counting of the RNA primer. The N-terminal half of Pri2 binds directly to Pri1 via a conserved protein-protein interface as revealed by a crystallographic study of the archeaon core primase (Lao-Sirieix et al., 2005). The Pri2 CTD (residues 307–528 of the *S. cerevisiae* protein), which is absent in the core Pri1-Pri2 crystal structure, contains a [4Fe-4S] cluster that is coordinated by four conserved cysteine ligands (Klinge et al., 2007; Sauguet et al., 2010). Biochemical studies of recombinant Pri2 proteins show that individual cysteine substitutions cause only moderate reduction of Fe binding and iron-sulfur (Fe-S) cluster content. In contrast, mutating two of the four cysteines nearly abolishes iron binding and significantly reduces initiation of RNA synthesis in an *in vitro* primase activity assay (Klinge et al., 2007). These studies were performed with proteins that are heterologously expressed in bacteria with a Fe-S assembly machinery equivalent to that of the mitochondrial iron-sulfur cluster (ISC) biogenesis pathway in eukaryotic cells. However, bacteria lack the eukaryotic cytosolic Fe-S assembly pathway and in some cases are unable to express eukaryotic proteins with native Fe-S cluster assembly (Urzica et al., 2009). Thus, the *in vivo* function of Pri2’s putative Fe-S cluster-binding domain and the relative contribution of the cysteine ligands in DNA replication initiation remain unclear.

Fe-S cluster-binding domains have been found in an increasing number of nuclear proteins that are involved in DNA replication and repair processes, including the helicases XPD/Rad3 and FancJ (Liu et al., 2008; Rudolf et al., 2006), the nucleases AddAB and Dna2 (Messick et al., 2002; Yeeles et al., 2009), and all four of the eukaryotic class B DNA polymerases: α, δ, ε and ζ (Baranovskiy et al., 2012; Netz et al., 2012). Biochemical and structural studies suggest that the Fe-S cluster binding domains in these nuclear enzymes serve a structural rather than a redox-active role, likely by stabilizing local domain conformation that may mediate protein-protein or protein-nucleic acid interactions. Nevertheless, the Fe-S clusters in some proteins such as yeast aconitases Aco1 and Lys4 are known to be targets of oxidative damage (Brazzolotto et al., 1999; Lu and Cortopassi, 2007; Missirlis et al., 2003; Wallace et al., 2004). Therefore, we were interested in investigating whether the Fe-S clusters in the DNA replication enzymes may also be susceptible to oxidative damage.

In this study, we characterize the *in vivo* role of the Fe-S cluster binding domain in Pri2 function by systematic site-directed mutagenesis of the putative cysteine ligands. We followed Fe-S assembly directly in yeast cells by ^55^Fe radiolabeling and determined the consequence of the cysteine mutations on S phase progression and pol-prim loading at early replication origins by chromatin immunoprecipitation (ChIP). We find that Fe-S cluster assembly in Pri2 is critical for maintaining its protein stability *in vivo* and is dependent on the function of Tah18, a diflavin NADPH reductase that is an essential component of the cytosolic iron-sulfur assembly (CIA) (Nunez-Ramirez et al.) machinery (Netz et al., 2010). Cysteine mutants of Pri2 exhibit temperature-sensitive growth defects. At the permissive temperature, the mutants are defective in ^55^Fe incorporation into nascent Pri2 polypeptide and exhibit reduced loading of multiple pol-prim subunits onto early replication origins. The Pri2 mutant proteins are further destabilized at the restrictive temperature, leading to permanent cell cycle arrest in the S phase with a G_1_ DNA content. We also find that Pri2 protein is unstable under iron-deficient growth conditions and in *sod1Δ* mutant cells lacking the cytosolic Cu/Zn superoxide dismutase. These findings demonstrate the importance of the conserved cysteine residues for Fe-S assembly into Pri2 and their essential role in maintaining the active pol-prim complex at replication origins. Our data also suggest that the initial step of DNA replication may be modulated by oxidative stress.

## Results

### Mutations in conserved cysteine residues of the Pri2 Fe-S cluster binding domain cause growth defect at restrictive temperatures

**Figure 1:**
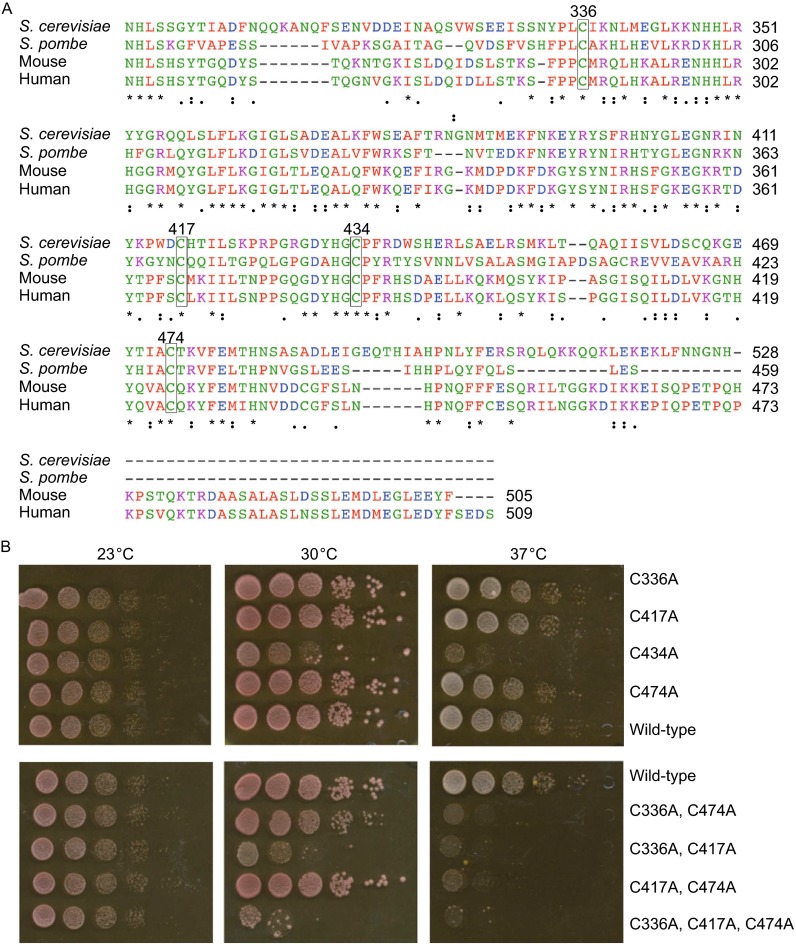
**Importance of conserved cysteine residues of Pri2’s C-terminal Fe-S cluster binding domain for mitotic viability**. (A) Multiple sequence alignment of primase large subunit C-terminal domains. Sequences are shown for *S. cerevisiae*, *S. pombe*, *H. sapiens* (Human), *M. musculus* (Mouse). The conserved cysteines were marked in boxes. The residue numbers are those of *S. cerevisiae* Pri2 protein. (B) Comparison of growth between congenic wild-type and *pri2* mutants with various Cys-to-Ala substitutions at different temperatures. Cells were grown in liquid YPD media to log phase (OD_600_ ~1) and harvested. Cell pellets were washed with water. Ten-fold serial dilutions starting at 10^5^ cells were dot-plated on YPD plates and incubated at 23°C, 30°C and 37°C for two days before being photographed

The four conserved cysteine residues in Pri2’s C-terminal Fe-S cluster binding domain are located at positions 336, 417, 434 and 474 (Fig. 1A). To determine the physiological importance of these residues, we first made single cysteine-to-alanine substitutions at each position and integrated both the wild-type and the mutant alleles into the chromosomal *PRI2* locus with an inframe triple HA epitope at the C-terminus. Three of the four single mutants had no apparent effect on cell fitness at growth temperatures ranging from 23°C to 37°C (Fig. 1B, top panel). In contrast, the *pri2*(*C434A*) single mutant exhibited severe growth defects at 30°C and 37°C. Interestingly, a mutation at the same residue, *C434Y*, was previously shown to cause lethality at 37°C arresting cells with large-budded morphology (Francesconi et al., 1991), a characteristic phenotype of mutants defective in S phase progression. Cys-434 thus appears to play a more critical role in stabilizing the Fe-S cluster and/or local protein conformation relative to the other three cysteines in Pri2’s CTD. We then made double and triple mutants between cysteines 336, 417 and 474. The triple mutant *pri2*(*C336A*, *C417A*, *C474A*) showed severe growth defects at 30°C and 37°C. All three double mutants are temperature-sensitive (ts) at 37°C, with *pri2*(*C336A*, *C417A*) showing a growth defect comparable to those of *pri2*(*C434A*) and *pri2*(*C336A*, *C417A*, *C474A*) even at 30°C (Fig. 1B, bottom panel). Together, these results indicate important but varying contributions of the four Fe-S ligand cysteines to Pri2 function and mitotic viability.

### Cysteine mutations reduce Pri2 protein stability and arrest cells in the S phase at the restrictive temperature

**Figure 2:**
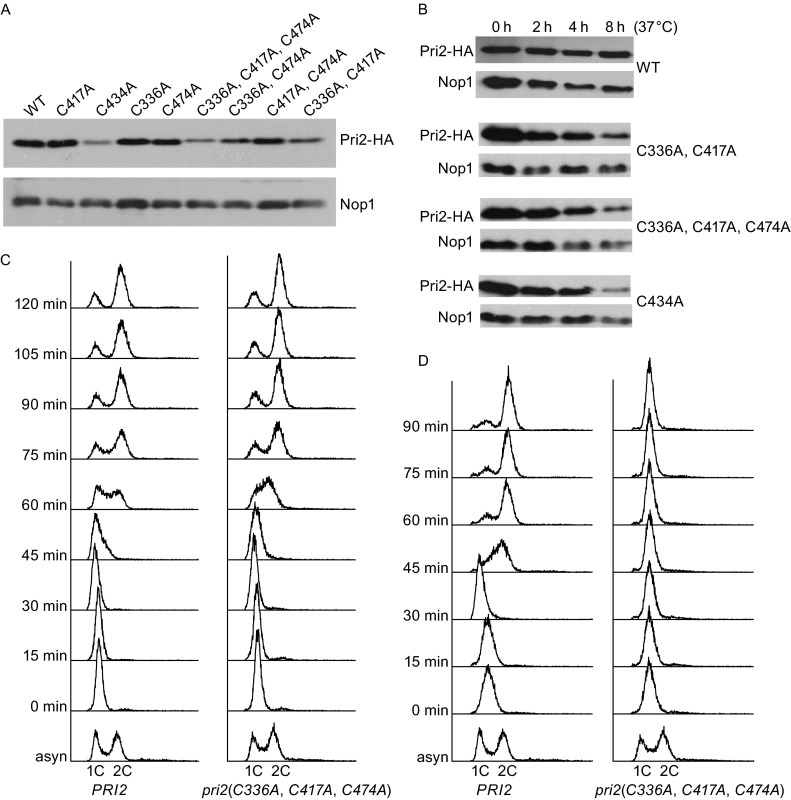
**Mutations of the cysteine residues of Pri2’s Fe-S cluster domain destabilize the Pri2 protein and impede DNA replication**. (A) Each *pri2* Cys-to-Ala mutant contained a C-terminal triple HA epitope and was integrated at the chromosomal *PRI2* locus. Congenic wild-type and *pri2* mutants were grown at 23°C to log phase. Protein extracts from harvested cells were resolved by SDS-PAGE and probed with anti-HA and anti-Nop1 (as a loading control). (B) Congenic wild-type and *pri2* mutants were grown at 23°C to log phase and then shifted to 37°C for an additional 2, 4 and 8 h. Protein extracts from harvested cells were subjected to immunoblotting for Pri2 and Nop1 proteins. Four-fold cell equivalents were loaded for each mutant relative to wild-type. (C and D) Comparison of cell cycle progression between wild-type (*PRI2*) and *pri2*(*C336A*,*C417A*, *C474A*) mutant cells at 23°C and 37°C. Cells were grown at 23°C to early log phase (OD_600_ ~0.3) and synchronized in G_1_ phase by using α factor. G_1_-arrested cells were split into two halves, released into cell cycle by washing out α factor at 23°C (C) and 37°C (D), and collected at the indicated time points for flow cytometry analysis

A couple of double cysteine substitutions in the Pri2 CTD have previously been shown to cause destabilization of the recombinant protein expressed in bacteria (Klinge et al., 2007). To assess the effect of the cysteine substitutions on Pri2 protein stability in yeast cells, we first compared Pri2 levels in log-phase cells between the wild-type and mutants at the permissive temperature (23°C). The *C434A* single, *C336A*, *C417A* double, and *C336A*, *C417A*, *C474A* triple mutants displayed significant decrease in Pri2 protein levels at 23°C (Fig. 2A). The three mutants also showed gradual destabilization of Pri2 protein when being shifted from 23°C to 37°C relative to the wild-type control (Fig. 2B), consistent with their growth defect at the restrictive temperature.

Mutants defective in DNA primase function are usually associated with DNA synthesis impairment and S phase delay (Longhese et al., 1993). To determine the contribution of the Pri2 Fe-S cluster binding domain to DNA replication, we compared the *pri2*(*C336A*, *C417A*, *C474A)* triple mutant and wild-type cells in progression through the first cell cycle after being released from an α factor-mediated G_1_-arrest by using flow cytometry. Both the wild-type and the mutant moved through the first S phase with similar pace after being released from G_1_ at 23°C (Fig. 2C). Upon being released at 37°C, the wild-type cells moved through the first S phase at a faster pace compared to 23°C (Fig. 2D). In contrast, the *pri2(C336A*, *C417A*, *C474A)* mutant permanently arrested with a G_1_ DNA content after being released from G_1_ at 37°C, indicative of a severe defect in initiation of DNA replication. The arrested unsynchronized mutant cells were mostly dumbbell shaped (data not shown), consistent with an S phase-arrested morphology. Taken together, these results suggest that disruption of Pri2’s Fe-S domain causes destabilization of the Pri2 protein, which impairs the initial step of DNA synthesis resulting in cell cycle arrest in S phase with a G_1_ DNA content.

### The pri2 cysteine mutants impair loading of the DNA Pol α-primase complex onto early replication origins

The growth defects and cell cycle arrest of the *pri2* cysteine mutants at the restrictive temperature suggested a critical role of its Fe-S cluster binding domain in replication initiation. A possible explanation is that lower stability and disturbance of local conformation of the *pri2* mutant proteins compromise loading of the pol-prim complex onto replication origins. To test this notion, we monitored association of individual pol-prim subunits with two well characterized early replication origins ARS305 and ARS607 by using chromatin immunoprecipitation (ChIP) and real-time PCR in *pri2*(*C336A*, *C417A*, *C474A*) and *pri2*(*C434A*), the two mutants with the most severe ts defect. The strains harbor *PRI2-HA* and *PRI1-Myc* or *POL1-Myc* that were integrated at their respective chromosomal loci. Cells from log-phase cultures were synchronized in G_1_ phase with α factor and then released into the cell cycle by washing out the α factor at 23°C. The relative enrichment of ARS DNA in the ChIP over input samples was used to evaluate change in association between various pol-prim subunits and the replication origins at different time points after release from G_1_.

**Figure 3:**
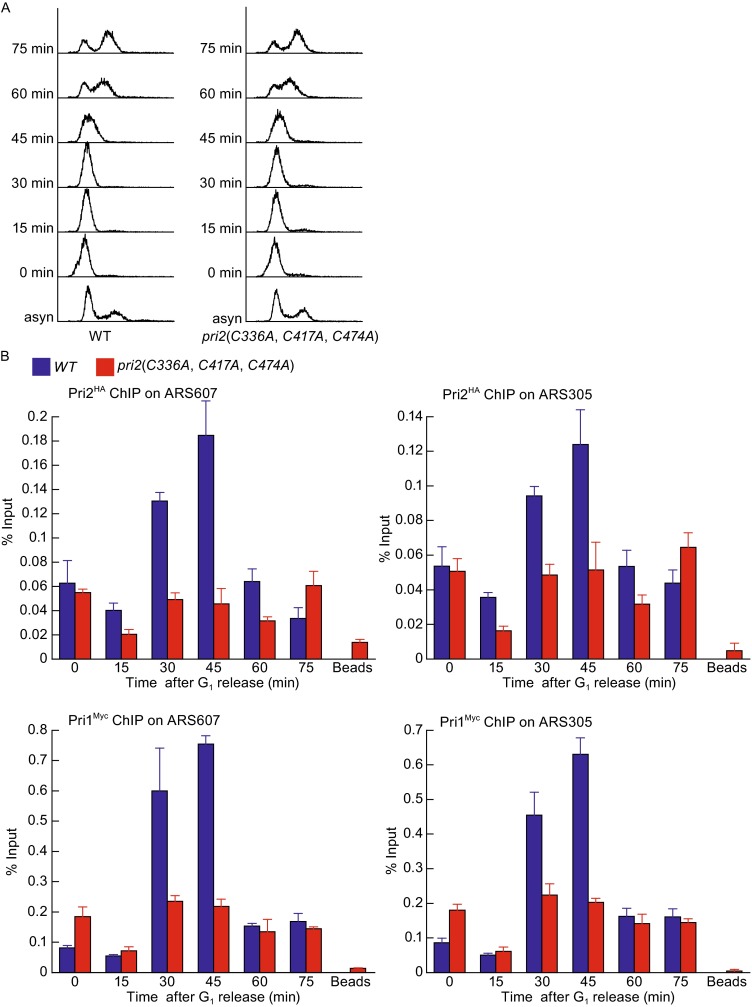
**Impaired loading of both DNA primase subunits onto early replication origins in the** ***pri2*** **(** ***C336A***, ***C417A***, ***C474A*** **)** **mutant**. Congenic wild-type (*PRI1-Myc*, *PRI2-HA*) and mutant (*PRI1-Myc*, *pri2*(*C336A*, *C417A*, *C474A)-HA*) cells were synchronized in G_1_ with α factor and released at 23°C. Cells were collected at the time points indicated. (A) FACS analysis indicates the DNA content of cells throughout the time course. (B) Chromatin-containing extracts were prepared from formaldehyde cross-linked cells collected at the indicated time points. Pri2-HA and Pri1-Myc were immunoprecipitated with anti-HA and anti-Myc monoclonal antibodies, respectively. The recovery efficiency of two early chromosomal replication origins, ARS305 and ARS607, in the immunoprecipitated material relative to the input material was determined by real-time PCR. Background was determined by calculating the amount of target DNA in the mock-IP sample relative to the Input sample (Beads). The results are an average of three independent experiments with standard deviations

In the wild-type cells, association of Pri1 and Pri2 at the two early origins was low in G_1_ and peaked at 30–45 min after G_1_ release (Fig. 3B, dark blue columns), which was before the bulk of DNA replication (based on FACS analysis, Fig. 3A). Dissociation of Pri1 and Pri2 from the two early origins coincided with a further increase of DNA bulk synthesis at the 60 min and 75 min time points. The temporal changes of the two primase subunits at ARS305 and ARS607 are consistent with previously observed kinetics of pol-prim complex loading at the two origins (Aparicio et al., 1999; Ricke and Bielinsky, 2004). In contrast, loading of Pri1 and Pri2 at the two early origins remained low in the *pri2*(*C336A*, *C417A*, *C474A*) mutant cells throughout the time course after G_1_ release, lacking any significant peak (Fig. 3B).

**Figure 4:**
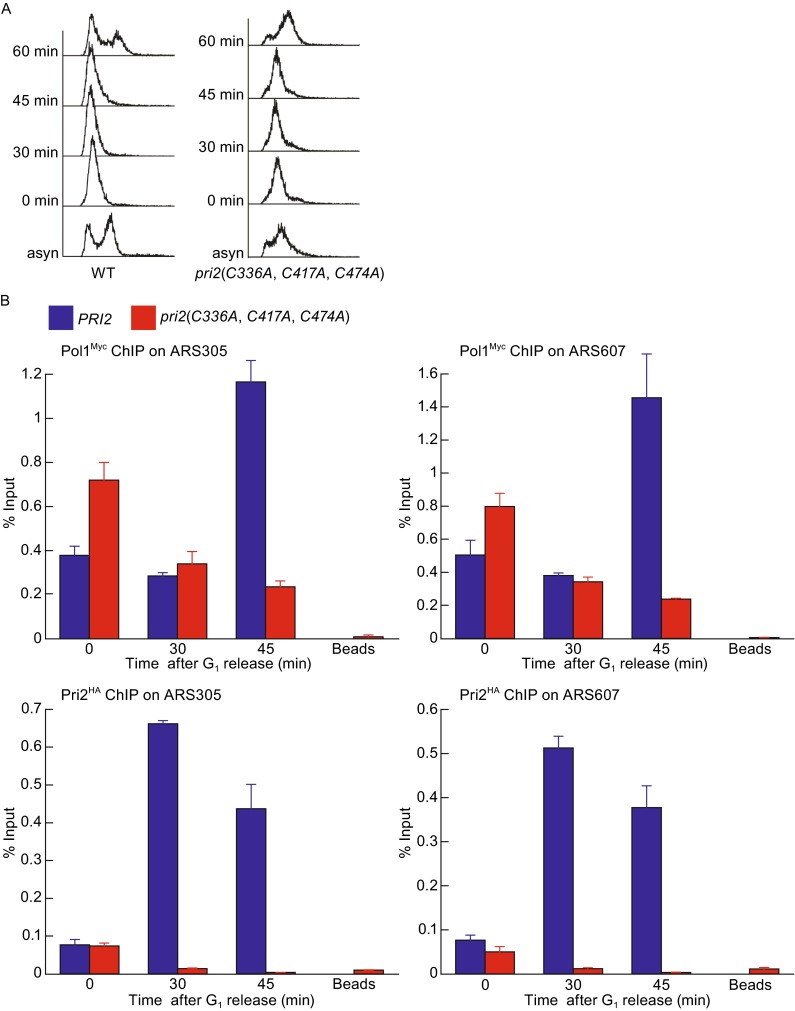
**Destabilized association between DNA polymerase α and early replication origins in the** ***pri2*** **(** ***C336A***, ***C417A***, ***C474A*** **)** **mutant**. Congenic wild-type (*POL1-Myc*, *PRI2-HA*) and mutant (*POL1-Myc*, *pri2*(*C336A*, *C417A*, *C474A*)*-HA*) cells were synchronized in G_1_ with α factor and released at 23°C. Cells were collected at the indicated time points for FACS (A) and ChIP (B) analyses as described in Fig. 3. Pol1- and Pri2-associated chromatin was immunoprecipitated using anti-Myc and anti-HA monoclonal antibodies, respectively

**Figure 5:**
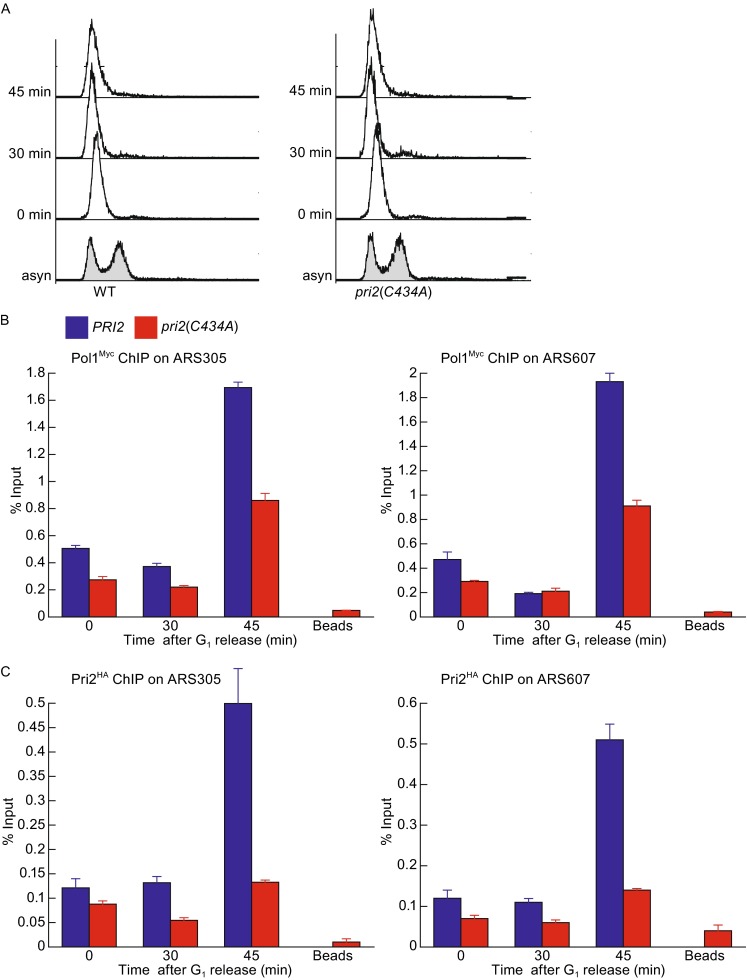
**Defective loading of Pri2 and Pol α onto early replication origins in the** ***pri2*** **(** ***C434A*** **)** **mutant**. Congenic wild-type (*POL1-Myc*, *PRI2-HA*) and mutant (*POL1-Myc*, *pri2*(*C434A*)*-HA*) cells were synchronized in G_1_ with α factor and released at 23°C. Cells were collected at the indicated time points for FACS (A) and ChIP (B) analyses as described in Fig. 3. Pol1- and Pri2-associated chromatin was immunoprecipitated using anti-Myc and anti-HA monoclonal antibodies, respectively

We next asked whether loading of DNA Pol α to the early replication origins was also affected in the *pri2* mutant. Since the pol-prim complex contains two heterodimers with distinct biochemical activities, it is unknown whether origin association of Pol1-Pol12 is independent of that of Pri1-Pri2. We found that there is a significant reduction of Pol1 association with ARS305 and ARS607 in *pri2*(*C336A*, *C417A*, *C474A*) mutant cells during the first 45 min after G_1_ release (Fig. 4). It is not clear the extent to which the relatively higher level of Pol1 binding at the origins at time zero reflects initial loading of Pol1, which may later be destabilized because of the failure of an increased loading of Pri1-Pri2. Similar to *pri2*(*C336A*, *C417A*, *C474A*), loading of Pri2 and Pol1 at the early origins were both defective in the *pri2*(*C434A*) mutant (Fig. 5). Since we have not observed any difference in levels of the two proteins between the wild-type and *pri2* mutants at 23°C (Fig. S1), the decreased loading of Pri1 and Pol1 at the two origins is unlikely due to variation in the endogenous protein levels. The results support the notion that decreased protein levels and origin loading of Pri2 can lead to destabilization of the whole pol-prim complex at the origins.

### The pri2 cysteine mutants are defective in Fe-S cluster assembly *in vivo*

**Figure 6:**
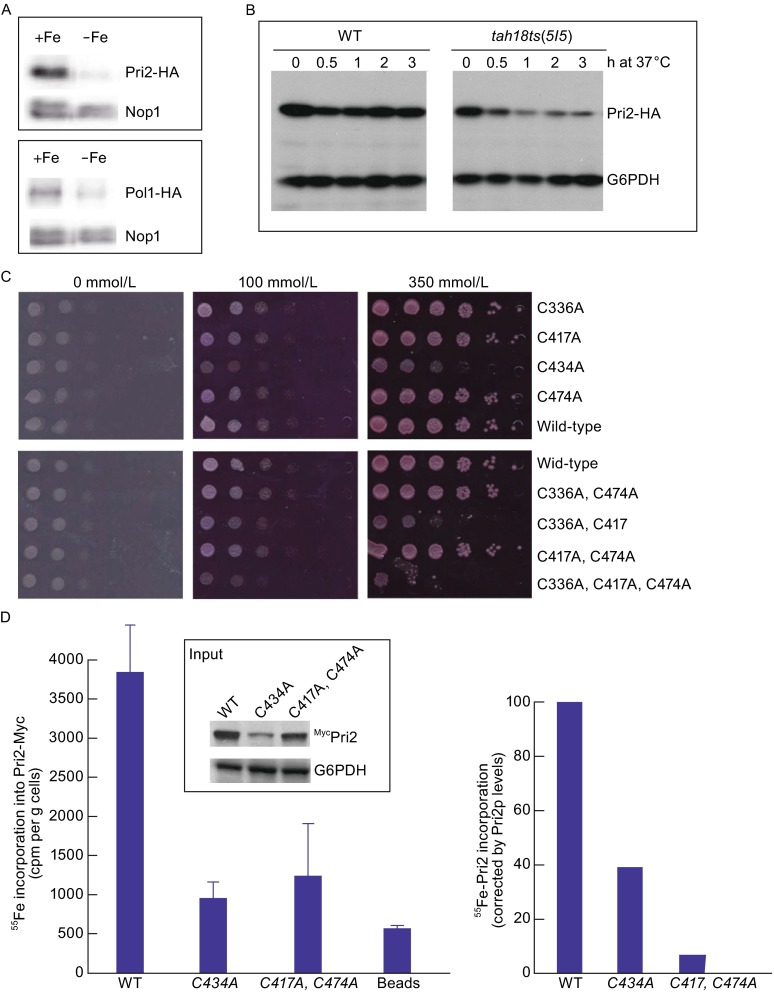
**Importance of Pri2 C-terminal conserved cysteine residues for** ***de novo*** **iron loading into the nascent Pri2 protein**. (A) Decreased Pri2 and Pol1 protein levels in cells grown under iron-deficient conditions. Wild-type cells containing integrated *PRI2-HA* or *POL2-HA* at the respective chromosomal loci were grown in regular Fe-supplemented SC media (+Fe) or in iron-depleted SC medium to log-phase before being harvested for protein extraction. Proteins were resolved by SDS-PAGE and probed with anti-HA for Pri2-HA and anti-Nop1 as a loading control. (B) Decreased Pri2 protein levels in the *tah18-5I5* mutant at the restrictive temperature. Both the wild-type and *tah18-5I5 ts* mutant contained C-terminal triple HA epitope integrated at the chromosomal *PRI2* locus. Cells were grown to log phase at 23°C before being shifted to 37°C at time zero. Protein extracts from cells collected at the indicated time points at 37°C were resolved by SDS-PAGE and probed with anti-HA for Pri2-HA and anti-G6PDH as a loading control. (C) The growth defects of *pri2* cysteine mutants are independent of Fe levels in media. Congenic wild-type and various *pri2* Cys-to-Ala mutants were dot-plated in 10-fold serial dilutions starting at 10^5^ cells onto selective media containing 1 mmol/L ferrozine (maximal ferrous chelating capacity at 333 μmol/L) and supplemental ferrous ammonium sulfate at 0 μmol/L, 100 μmol/L (iron-poor) and 350 μmol/L (iron-rich). The plates were incubated at 30°C for 2 days before being photographed. (D) The Pri2 cysteine mutants are defective in iron incorporation into newly translated Pri2 polypeptide. Wild-type cells that harbor a high-copy number vector (pRS426-P_TDH3_) expressing the Myc-tagged wild-type (pLL98) or *C434A* (pLL108) and *C417A*, *C474A* (pLL112) mutant Pri2 proteins were grown in synthetic iron-poor medium supplemented with 24 μmol/L BPS for 16 h at 30°C. Radiolabeling with ^55^Fe was conducted for 4 h at 30°C in a BPS-free medium and Myc-Pri2 proteins were immunoprecipitated from cell extracts using an anti-Myc monoclonal antibody. The radioactivity associated with Pri2 was quantified by scintillation counting (left panel) and corrected for the differences in protein levels (right panel), which were determined by immunoblotting and quantitative densitometry (insert)

Previous biochemical studies have shown that some substitutions of the four conserved cysteines in Pri2 CTD disrupted Fe-S assembly in the recombinant protein and hindered Pri2 purification because of protein destabilization (Klinge et al., 2007). One caveat is the recombinant protein may lack the native Fe-S cluster (Urzica et al., 2009). We sought to follow Pri2 Fe-S cluster assembly in yeast cells. The level and activity of some iron-containing proteins are known to be altered under iron-limiting growth conditions (Kaplan et al., 2006). Indeed, we show that protein levels of Pri2 and Pol1, both containing a Fe-S cluster binding domain, were markedly reduced in cells grown in iron-deficient medium in comparison to iron-replete medium (Fig. 6A).

We then examined whether deficiencies in the CIA machinery would have an effect on Pri2 protein levels by using a ts mutant of *TAH18*. Tah18 is a diflavin NADPH reductase that forms a complex with the Fe-S cluster protein Dre2 and together constitutes an essential and early step in cytosolic Fe-S protein biogenesis. Depletion of Dre2-Tah18 results in reduction of Fe-S assembly into many CIA substrates and subsequent destabilization of some of the apo-proteins (Netz et al., 2010). While Pri2 protein levels remained relatively unchanged between 23°C and 37°C in wild-type cells, we observed significant reduction in Pri2 levels when the *tah18-ts* mutant was shifted from 23°C to 37°C (Fig. 6B). These results suggest that Pri2 is a substrate of CIA and that the apo form of Pri2 is unstable *in vivo*.

We also compared growth of wild-type and different *pri2* cysteine mutants on synthetic medium containing 1 mmol/L of iron chelator ferrozine, which has maximum chelating capacity of 333 μmol/L ferrous iron (Stearman et al., Ö 1996), and varied concentrations of supplemental ferrous ammonium sulfate at 30°C. Of the eight mutants, *pri2*(*C434A*), *pri2*(*C336A*, *C417A*) and *pri2*(*C336A*, *C417A*, *C474A*) exhibited growth defects on iron-deprived medium (0, 100 μmol/L) relative to the wild-type and other single and double cysteine mutants (Fig. 6C). These were the same three mutants that exhibited the most severe ts growth defects on rich medium (Fig. 1B). The defect of the three mutants was not rescued by growth on an iron-rich (350 μmol/L) medium (Fig. 6C), suggesting that Fe-S cluster assembly in these mutants is defective, which cannot be remedied by an increase in ferrous iron supply. To directly test this hypothesis, we used ^55^Fe radiolabeling and immunoprecipitation assay to measure and compare iron incorporation into newly synthesized Pri2 proteins. Both the wild-type and two mutant Pri2(*C434A* and *C417A*, *C474A*) were N-terminally tagged with a triple Myc epitope and expressed from a high copy number plasmid to improve signal to background ratios of ^55^Fe labeling. Cells were grown in iron-free medium at 30°C overnight before being pulse-labeled with ^55^FeCl_3_ for 4 h. Pri2 were immunoprecipitated from whole cell extracts and the associated radioactivity was quantified by scintillation counting. Significantly more ^55^Fe were bound to the wild-type Pri2 protein relative to the two cysteine mutants. After corrected for differences in protein stability, the *C434A* and *C417A*, *C474A* mutant proteins had a 60% and 90% reduction in ^55^Fe binding, respectively (Fig. 6D). Together, these results demonstrate that Pri2 contains a *bona fide* Fe-S cluster *in vivo* that are coordinated by the conserved cysteine residues in its CTD. Our data also strongly suggest that the apo-form of Pri2 is unstable *in vivo*, which underlies the observed pol-prim loading defect at the permissive temperature and the replication initiation defect at the restrictive temperature.

### Fe-S cluster assembly into Pri2 is sensitive to oxidative stress

**Figure 7:**
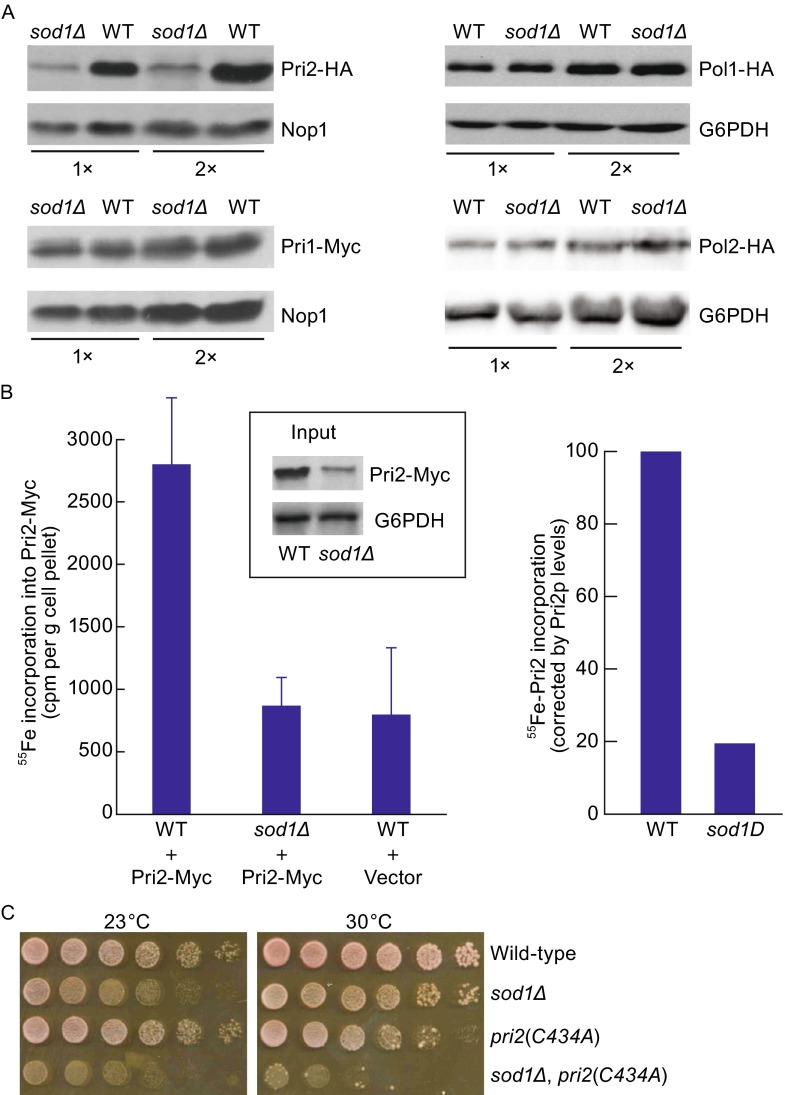
**Impaired Pri2 protein stability and iron binding in** ***sod1Δ*** **mutant cells**. (A) Comparison of protein levels of Pri2, Pri1 and Pol1 between log-phase congenic wild-type and *sod1∆* mutant cells. Pri2-HA, Pri1-Myc, Pol1-HA and Pol2-HA were expressed from their respective chromosomal loci under the endogenous promoters. Protein extracts were subjected to immunoblotting using monoclonal anti-HA and anti-Myc antibodies. Nop1 and G6PDH were probed as loading controls. (B) Fe-S cluster assembly on Pri2 protein is compromised in *sod1∆* mutant. Congenic wild-type and *sod1∆* cells harboring pRS426-P_TDH3_-Myc-Pri2 were grown and treated as described in the legend of Fig. 5B except for that radiolabeling with ^55^Fe was conducted for 2 h. The amount of ^55^Fe bound to immunoprecipitated Pri2 was quantified by scintillation counting (left panel) and corrected for the differences in Pri2 protein levels between wild-type and *sod1∆* cells (right panel), as determined by immunoblotting and quantitative densitometry (insert). (C) Synthetic lethality between *sod1∆* and *pri2*(*C434A*) mutant alleles. Congenic wild-type (LLY263), *pri2*(*C434A*) (LLY260), *sod1∆* (LLY338) and *pri2*(*C434A*), *sod1∆* (LLY427) mutant cells were harvested from log phase cultures and dot-plated on YPD medium in 10-fold serial dilutions starting at 10^5^ cells. The plates were incubated at 23°C and 30°C for 2 days before being photographed

The redox chemistry of Fe-S clusters means they are inherently vulnerable to oxidation and degradation. Superoxide has been shown to oxidize exposed [4Fe-4S] clusters in some proteins such as aconitase (Aco1) and isopropylmalate isomerase (Leu1), causing loss of iron from the cluster and inactivation of the enzyme (Djaman et al., 2004). Since both Pri2 and replicative DNA polymerases contain a [4Fe-4S] cluster, we were interested in determining whether the cluster assembly and stability of these proteins are affected by increased oxidative stress in the *sod1Δ* mutant lacking the Cu/Zn superoxide dismutase. We observed a significant decrease of Pri2 protein levels in *sod1Δ* relative to the wild-type cells. In contrast, there was no difference in protein levels of Pri1 between wild-type and *sod1Δ* (Fig. 7A). The proteins levels of two other [4Fe-4S] containing replication enzymes Pol1 and Pol2 were not affected by *sod1Δ* either. It thus seems that Pri2 is more vulnerable to oxidative damage relative to other Fe-S containing proteins involved in DNA replication. Indeed, we find that Pri2 is further destabilized when *sod1* cells were treated with oxidants paraquat and hydrogen peroxide (Fig. S2).

To determine if Fe-S cluster assembly in Pri2 is sensitive to oxidative stress, we carried out ^55^Fe labeling and Pri2 immunoprecipitation assays in wild-type and *sod1Δ* mutant cells expressing transformed Myc-tagged Pri2. ^55^Fe incorporation into newly translated Pri2 is 5-fold lower in *sod1Δ* mutant relative to the wild-type control after being corrected for difference in Pri2 protein levels (Fig. 7B). The *sod1Δ* mutant has previously been shown to have a prolonged S phase (Carter et al., 2005). Our results suggest that impaired Fe-S cluster assembly into nascent Pri2 polypeptide and the resulted destabilization of the apo-Pri2 may contribute to the S phase defect in *sod1Δ* mutant. In keeping with this notion, we found that the *pri2*(*C4343A*) mutant allele exhibited synthetic growth defect with *sod1Δ*; the double mutant is lethal at 30°C while each single mutant showed only mild growth defect (Fig. 7C). The synthetic phenotype between *sod1* and *pri2-ts* points to a connection between oxidative stress and DNA replication initiation.

## Discussion

Precise temporal and spatial control of the initiation events is critical for optimal replication of the genome and maintenance of genomic stability (Casper et al., 2008; Foiani et al., 1997; Lemoine et al., 2008). The four-subunit pol-prim complex performs a unique function in the initial step of DNA synthesis. How the complex is assembled onto and maintained at the replication origins is not fully understood. In this report, we provide genetic and molecular evidence that the Pri2 Fe-S cluster domain is critical for stable association of the pol-prim complex with the origins during replication initiation. Mutations at the conserved cysteine residues that disrupt Fe-S cluster assembly in Pri2 destabilize the protein and reduce association of the entire pol-prim complex with the origins. We also show that Fe-S cluster assembly into newly synthesized Pri2 is sensitive to increased oxidative stress thus linking replication initiation to cellular redox state.

Our mutagenesis studies clearly show that the four conserved cysteine ligands of the Pri2 Fe-S cluster exhibit different roles in maintaining the Fe-S cluster and local protein conformation. Of the single cysteine substitutions, only *C434A* causes a severe growth defect and replication impairment at the restrictive temperature. Crystallographic analysis of the yeast Pri2 CTD reveals a larger N-terminal (residues 321–433) and a smaller C-terminal (residues 434–512) subdomains, which are bridged together with the Fe-S cluster situated at the seam with two cysteines from each subdomain acting as iron ligands (Sauguet et al., 2010). Cysteines 336, 417 and 474 are located within α-helices, which are unlikely to be disrupted by a Cys-to-Ala substitution. This structural organization is consistent with the normal growth phenotype of the three single mutants (Fig. 1B) and previously observed moderate loss of iron binding *in vitro* (Klinge et al., 2007). It is possible that a [3Fe-4S] cluster, likely resulted from a single cysteine substitution in Pri2, is sufficient to maintain local conformation and stability in the three single mutants. On the contrary, the unique position of Cys-434 at the linker region between the two subdomains suggests a more important role in maintaining the Fe-S cluster in place. In addition to destabilizing the Fe-S cluster, substitutions at Cys-434 may weaken shielding of the Fe-S cluster within a hydrophobic environment by exposing it to the aqueous environment, leading to Pri2 protein instability.

The Pri2 CTD has been implicated in association with several proteins involved in replication initiation. Although the archea Pri1 and Pri2 NTD form a stable complex *in vitro* (Lao-Sirieix et al., 2005), in the human Pri2 both the N- and C-terminal regions are involved in physical association with Pri1 (Copeland, 1997). Within the pol-prim complex, Pri2 binds Pol α providing a link between the primase and the DNA polymerase (Copeland and Wang, 1993; Longhese et al., 1993; Mizuno et al., 1999; Tan and Wang, 2000). A recent cryo-EM study suggests that Pri2’s CTD is in direct contact with Pol α (Nunez-Ramirez et al., 2011). The atomic structure of the human Pri2 CTD (p58C) reveals a strikingly basic surface suggesting DNA or RNA binding activity (Vaithiyalingam et al., 2010). In fact, the human p58C does bind nucleic acids *in vitro*, with a preference for ssRNA/dsDNA junction substrates (Vaithiyalingam et al., 2010). The human p58C also interacts with the ssDNA binding protein RPA, which may help recruiting primase to the origin and releasing RPA from the template ssDNA during priming step. It is conceivable that these interactions are compromised by mutations that disrupt the Fe-S cluster assembly and proper folding of the Pri2 CTD.

How the pol-prim complex is brought onto replication origins remains to be elucidated. Previous studies show that Mcm10 associates with and stabilizes Pol α regardless of the stage of the cell cycle (Ricke and Bielinsky, 2004; Ricke and Bielinsky, 2006). Thus, individual subunits of the pol-prim complex may exist as monomers independent of the other subunits at some stages of the cell cycle. The loading of Pol α to replication origins requires Mcm10 and Ctf4 (Zhu et al., 2007) while Pri1-Pri2 loading may involve RPA (Tanaka and Nasmyth, 1998). It is unclear whether the Pol1-Pol12 and Pri1-Pri2 heterodimers are recruited to the origins separately or as one concerted step. Nevertheless, our finding that Pri2 cysteine mutants disrupting origin association of Pri1-Pri2 also severely destabilize origin binding of Pol α strongly suggest that the interaction between the pol and prim heterodimers, likely mediated by Pri2, is critical in maintaining/stabilizing the entire pol-prim complex at the origins.

We find that some *pri2* cysteine mutants exhibit normal S phase progression pace at the permissive temperature even though the Pri2 protein level is much lower than the wild-type control. It would thus appear that the level of pol-prim subunit is not usually rate-limiting during S phase entry. Consistent with the notion, these mutants become permanently arrested with a G_1_ DNA content with further diminishing Pri2 protein levels when shifted to the restrictive temperature. Previous studies show that the Pol1 protein levels remain relatively unchanged throughout the cell cycle despite of periodic fluctuation in *POL1* mRNA levels (Muzi Falconi et al., 1993). The assembly of the pol-prim complex is not restricted to the S phase either (Ferrari et al., 1996). Nevertheless, lower pol-prim levels or weakened origin association that approaches below a minimal threshold may alter the temporal and spatial pattern of origin firing in S phase, as was shown for the key origin firing factors Cdc45 and Sld3 (Mantiero et al., 2011; Tanaka et al., 2011).

The recent discoveries of a Fe-S cluster binding domain in Pri2 and three major replicative DNA polymerases raise the possibility that DNA replication may be subjected to redox regulation. Crystal structure of the human Pri2 CTD shows that the Fe-S cluster is buried within the hydrophobic core, with very little exposure to the surface (Vaithiyalingam et al., 2010). Nevertheless, the Fe-S cluster may still be accessible to small molecules that may cause damage. Superoxide radical is known to be able to inactivate many [4Fe-4S] cluster-containing enzymes by oxidation of the cluster, leading to loss of the labile iron (Fridovich, 1995; Gardner and Fridovich, 1991; Longo et al., 1999). Our finding of decreased Fe-S assembly and protein stability of Pri2 in the *sod1Δ* mutant suggests that Pri2 may be susceptible to oxidative damage. An alternative explanation is that a diminished cytosolic pool of bioavailable iron in *sod1Δ* mutant (De Freitas et al., 2000; Srinivasan et al., 2000) may impair Fe-S assembly in Pri2. The protein levels of Pol1 and Pol2 (i.e. Pol α and Pol ε), both containing a Fe-S cluster, remain unchanged in *sod1Δ* mutant relative to the wild-type. Some apo proteins retain stability but lose activity when deprived of their Fe-S clusters (Kispal et al., 1999). Therefore, we cannot rule out the possibility of oxidative damage to the Fe-S clusters in Pol1 and Pol2 in *sod1Δ* mutant. The Fe-S cluster in Pri2 is likely more vulnerable to oxidative damage relative to Pol1. We propose that Pri2 is a key component of the replisome that acts to link regulation of replication initiation to changes in cellular redox state.

## Materials and Methods

### Yeast strains, cell growth and plasmids

Yeast strains and plasmids used in this study were listed in Tables 1 and 2, respectively. All yeast strains were derived from a W303 parental strain Y300 except for LLY277 (*8C2*, *PRI2-HA*) and LLY278 (*tah18-5I5*, *PRI2-HA*), which were generated by integrating *PRI2-3HA* into the chromosomal *PRI2* locus in strains 8C2 and 5I5 (Vernis et al., 2009). Epitope-tagging, promoter replacement and gene deletion were constructed by PCR-based homologous recombination method as described earlier (Longtine et al., 1998). Substitutions of Pri2’s Fe-S domain conserved cysteine residues were introduced by using the QuikChange site-directed mutagenesis kit (Stratagene) according to manufacturer’s instruction. DNA sequencing was used to verify the desired sequences of all mutant *PRI2* alleles.

**Table 1:** **Yeast strains used in this study**

Strain	Genotype	Parental strain
Y300	*MATa*, *can1-100*, *ade2-1*, *his3-11*,*15*, *leu2-3*,*112*, *trp1-1*, *ura3-1*	
LLY263	*MATa*, *pri2*::*PRI2-3HA-TRP1*, *bar*::*LEU2*	Y300
LLY271	*MATa*, *pri2*::*PRI2-C417A-3HA-TRP1*, *ba1r*::*LEU2*	Y300
LLY260	*MATa*, *pri2*::*PRI2-C434A-3HA-TRP1*, *bar1*::*LEU2*	Y300
LLY378	*MATa*, *pri2*::*PRI2-C336A-3HA-TRP1*, *bar1*::*LEU2*	Y300
LLY366	*MATa*, *pri2*::*PRI2*-*C474A-3HA-TRP1*, *bar1*::*LEU2*	Y300
LLY368	*MATa*, *pri2*::*PRI2*-*C336A*,*C417A*,*C474A-3HA-TRP1*, *ba1r*::*LEU2*	Y300
LLY360	*MATa*, *pri2*::*PRI2*-*C336A*,*C474A-3HA-TRP1*, *bar1*::*LEU2*	Y300
LLY288	*MATa*, *pri2*::*PRI2*-*C417A*,*C474A-3HA-TRP1*, *bar1*::*LEU2*	Y300
LLY292	*MATa*, *pri2*::*PRI2*- *C336A*,*C417A-3HA-TRP1*, *bar1*::*LEU2*	Y300
LLY343	*MATa, pri2*::*PRI2-3HA-TRP1, pri1*:: *PRI1-13Myc-HIS5*, *bar1*::*LEU2*	Y300
LLY372	*MATa, pri2*::*PRI2*-*C336A*,*C417A*,*C474A-3HA-TRP1*, *pri1*::*PRI1-13Myc-HIS5*, *bar1*::*LEU2*	Y300
LLY382	*MATa*, *pri2*::*PRI2-3HA-TRP1*, *pol1*::*POL1-13Myc-HIS5*, *bar1*::*LEU2*	Y300
LLY387	*MATa*, *pri2*::*PRI2*-*C336A,C417A*,*C474A-3HA-TRP1*, *pol1*::*POL1-13Myc-HIS5*, *bar1*::*LEU2*	Y300
LLY328	*MATa*, *pri2*::*PRI2-3HA-TRP1*, *pri1*::*PRI1-13Myc-HIS5*, *bar1*::*LEU2, sod1*::*KAN*	Y300
LLY330	*MATa*, *pri2*::*PRI2-3HA-TRP1*, *pri1*::*PRI1-13Myc-HIS5*, *bar1*::*LEU2*	Y300
LLY398	*MATa*, *pol1*::*POL1-3HA-TRP1*, *bar1*::*LEU2*	Y300
LLY399	*MATa*, *pol1*::*POL1-3HA-TRP1*, *bar1*::*LEU2*, *sod1*::*KAN*	Y300
LLY333	*MATa*, *sod1*::*KAN*	Y300
LLY338	*MATa*, *bar1*::*LEU2*, *sod1*::*KAN*	Y300
LLY426	*MATa*, *bar1*::*LEU2*	Y300
LLY427	*MATa*, *bar1*::*LEU2*, *sod1*::*KAN*, *pri2*::*PRI2-C434A-3HA-TRP1*	Y300
LLY428	*MATa*, *bar1*::*LEU2*, *sod1*::*KAN*, *pri2*::*PRI2*-*C336A*,*C417A*,*C474A-3HA-TRP1*	Y300
LLY277	*MATα*, *ura*,*3 leu2*, *trp*,*1 lys2*, *cyh2* ^*R*^, *PRI2-HA*	8C2 (Vernis et al., 2009)
LLY278	*MATα*, *ura3 leu2*, *trp1*, *lys*,*2 cyh2* ^*R*^, *PRI2-HA*, *tah18-5I5*	5I5 (Vernis et al., 2009)

**Table 2:** **Plasmids used in this study**

Plasmid	Description	Reference
pLL112	pRS426-P_TDH3_-3Myc-PRI2-C417,474A	This study
pLL108	pRS426-P_TDH3_-3Myc-PRI2-C434A	This study
pLL98	pRS426-P_TDH3_-3Myc-PRI2	This study
PRI2 (WT)	pRS404-Pri2-CTD (residues 201–528)-3HA	Ricke and Bielinsky, 2006
PRI2 (C336A)	pRS404-Pri2-CTD-C336A-3HA	This study
PRI2 (C417A)	pRS404-Pri2-CTD-C417A-3HA	This study
PRI2 (C434A)	pRS404-Pri2-CTD-C434A-3HA	This study
PRI2 (C474A)	pRS404-Pri2-CTD-C474A-3HA	This study
PRI2 (C336A/C474A)	pRS404-Pri2-CTD-C336A,C474A-3HA	This study
PRI2 (C336A/C417A)	pRS404-Pri2-CTD-C336A,C417A-3HA	This study
PRI2 (C474A/C417A)	pRS404-Pri2-CTD-C474A,C417A-3HA	This study
PRI2 (C336A/C417A/C474)	pRS404-Pri2-CTD-C336A,C417A,C474-3HA	This study

Rich medium (YPD) contained 1% yeast extract, 2% peptone and 2% glucose. Synthetic complete (SC) medium contained 0.17% yeast nitrogen base without amino acids and (NH_4_)_2_SO_4_ (MP Biomedicals), 0.5% (NH_4_)_2_SO_4_, all twenty amino acids (Sigma) at concentrations as described (Burke et al., 2000) and 2% of glucose. Selective (i.e., drop-out) media were SC omitting one or multiple amino acid as indicated. For solid media, 2% Bacto Agar was added before autoclaving. Iron-buffered medium consisted of modified synthetic complete medium to which MES (50 mmol/L 2-(N-morpholino)ethanesulfonic acid, pH 6.1) buffer and iron chelator (1 mmol/L ferrozine) were added. Different amounts of ferrous ammonium sulfate (0, 100, 350 μmol/L) were added back to establish windows of available ferrous iron as described earlier (Stearman et al., 1996).

### Protein extraction and immunoblotting

Yeast cells were harvested from early- to mid-log phase cultures (OD_600_ 0.5~1.0). Protein extracts were prepared by using the trichloroacetic acid method as described earlier (Wu et al., 2011). Proteins were resolved by 8%–12% SDS-PAGE, transferred to nitrocellulose membranes, and probed with primary and secondary antibodies. Blots were developed with an enhanced chemiluminescence substrate (Perkin-Elmer). Primary antibodies were used at the following dilutions: monoclonal anti-HA 12CA5 (Roche) at 1:10,000, monoclonal anti-Myc 9E10 (Roche) at 1:2,000, monoclonal anti-Nop1 (EnCor Biotechnology) at 1:2,500 and anti-G6PDH (Sigma) at 1:200,000.

### Cell cycle synchronization and flow cytometry

For α factor block and release experiments, yeast cells (all *bar1Δ* mutants) were harvested from a log-phase culture, re-suspended in fresh YPD containing 300 nmol/L of α factor at a final OD_600_ of 0.3, and incubated at for 3–4 h at the indicated temperatures until >95% of the cells were arrested in G_1_. Cells were released from G_1_ arrest by washing out the α factor with fresh YPD containing 0.1 mg/mL pronase E (Sigma) at the indicated temperatures and harvested at different time points. DNA content was measured as described earlier (Wu et al., 2011) with a Beckman Coulter Epics XL-MCL flow cytometer and DeltaGraph software.

### Chromatin immunoprecipitation (ChIP) and Real-Time PCR analyses

ChIP was performed as described earlier (Aparicio et al., 2004). Fifty mL of cells (2.0 × 10^7^ cells/mL) were treated with formaldehyde to cross-link proteins to DNA. Cells were lysed by glass beads disruption in 500 μL FA-lysis buffer (50 mmol/L HEPES-KOH at pH 7.5, 140 mmol/L NaC1, 1 mmol/L EDTA, 1% Triton X-100, 0.1% sodium deoxycholate) supplemented with 1 mmol/L PMSF and protease inhibitor cocktail (Roche). The suspension was sonicated with a Bioruptor (Diagenode) for 40 min (30 sec on/off) each, resulting in an average fragment size of 0.3~0.5 kb). DNA bound to HA and Myc-tagged Pri2, Pri1 and Pol1 was immunoprecipitated with anti-HA (12CA5) and anti-Myc (9E10), respectively, at 1:100 *v*/*v* ratio from 2 mg of lysates at 4°C overnight. One-tenth of the lysate was set aside as input. Precipitates were successively washed for 15 min each with FA-lysis buffer, FA-lysis buffer with 500 mmol/L NaCl, LiCl buffer (10 mmol/L Tris-HCl at pH 8.0, 0.25 mol/L LiC1, 0.5% NP-40, 0.5% sodium deoxycholate, 1 mmol/L EDTA) and TE (20 mmol/L Tris-HC1 at pH 8.0, 1 mmol/L EDTA) and eluted by incubation in TE with 1% SDS at 65°C for 15 min. The input and immunoprecipitated samples were then processed for DNA purification as described (Aparicio et al., 2004). Finally, the DNA was purified using a QiaQuick PCR purification kit (Qiagen).

The DNA was diluted (1:3 for immunoprecipitated DNA and 1:30 for input DNA) and used in quantitative real-time PCR using the Bio-Rad CFX96 detection system based on SYBR Green fluorescence according to the manufacturer’s protocol. PCR reactions were performed in triplicate on 96-well plates, and an average cycle threshold values was calculated for each reaction. The amount of target DNA in the ChIP sample relative to the Input sample was calculated. Background was determined by calculating the amount of target DNA in the mock-IP sample relative to the Input sample. The primer pair for amplification of the region surrounding ARS305 was 5′-TTTGGAGCTCAAGTGGATTG-3′ and 5′-CACACCGGACAGTACATGAAA-3′. The primer pair for amplification of the region surrounding ARS607 was 5′-CATTTACGCACTCTAACTGGC-3′ and 5′-AAACCAATAGCAGGATCGACC-3′.

### ^55^Fe labeling and immunoprecipitation of Pri2

 Radiolabeling of yeast cells with ^55^FeCl_3_ (Perkin-Elmer) and measurement of ^55^Fe incorporation into proteins by immunoprecipitation were performed as described earlier (Zhang et al., 2011). The standard iron-depleted medium was supplemented with 24 μmol/L bathophenanthrolinedisulfonate (BPS) to chelate any extra ferrous iron in order to make iron-free medium. Wild-type cells harboring a 2-micron plasmid (pRS426-P_TDH3_) that overexpresses N-terminally Myc-tagged Pri2 (pLL98), Pri2-C434A (pLL108), or Pri2-C417A, C474A (pLL112) mutant proteins were grown in the iron-free medium to log-phase and harvested. For each sample, 1–2 × 10^9^ cells (wet weight 0.1–0.2 g) were labeled with 20 μCi of ^55^FeCl_3_ in for 4 h at 30°C. Wild-type and mutant Myc-Pri2 proteins were immunoprecipitated from 2 mg of whole cell lysates using monoclonal anti-Myc (9E10) at 1:100 *v*/*v* ratio. Pri2-bound ^55^Fe in the immunoprecipitates were measured by scintillation counting.

## Electronic supplementary material

Below is the link to the electronic supplementary material.

Supplementary material 1 (PDF 128 kb)

Supplementary material 2 (PDF 172 kb)

## Abbreviations

ChIP: chromatin immunoprecipitation
CIA: cytosolic iron-sulfur cluster protein assembly
Fe-S: iron-sulfur
pol-prim: DNA polymerase α-primase
