# Supplementary material for: Essential role of the iron-sulfur cluster binding domain of the primase regulatory subunit Pri2 in DNA replication initiation
Source: Protein Cell. 2015 Feb 4;6(3):194–210. doi: 10.1007/s13238-015-0134-8 (PMC4348247; doi:10.1007/s13238-015-0134-8)
Supplement: Supplementary file 1 — Supplementary material 1 (PDF 128 kb) [file 13238_2015_134_MOESM1_ESM.pdf]

Figure S1

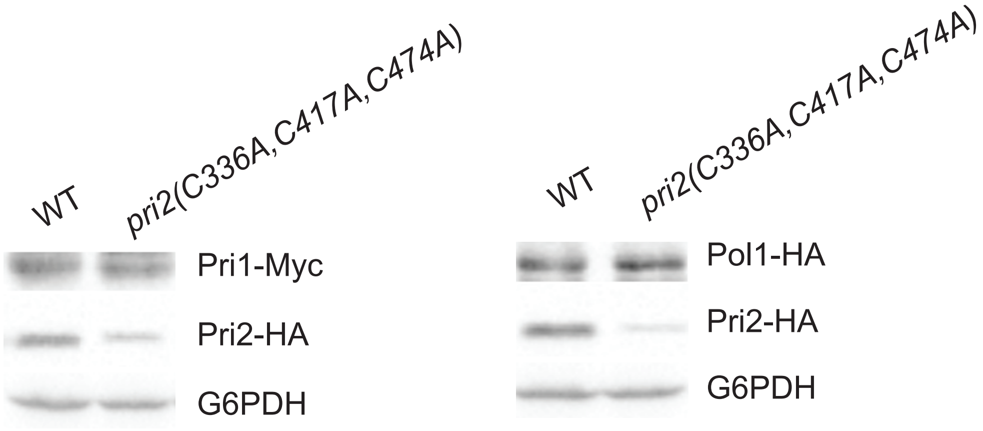

Supplemental Figure S1. Protein levels of Pri1 and Pol1 protein remain unchanged in *pri2(C336A,C417A,C474A)* mutants.

Protein extracts from log-phase cells were resolved by SDS-PAGE and probed for the epitope-tagged proteins and G6PDH as a loading control. (A) Both wild-type (WT) and pri2(C336A,C417A,C474A) contained PRI1-MYC and PRI2-HA at their respective chromosomal loci. (B) Both wild-type (WT) and pri2(C336A,C417A,C474A) contained POL1-HA (171 kD) and PRI2-HA (67 kD) at their respective chromosomal loci.
