# Supplementary material for: Essential role of the iron-sulfur cluster binding domain of the primase regulatory subunit Pri2 in DNA replication initiation
Source: Protein Cell. 2015 Feb 4;6(3):194–210. doi: 10.1007/s13238-015-0134-8 (PMC4348247; doi:10.1007/s13238-015-0134-8)
Supplement: Supplementary file 2 — Supplementary material 2 (PDF 172 kb) [file 13238_2015_134_MOESM2_ESM.pdf]

Figure S2

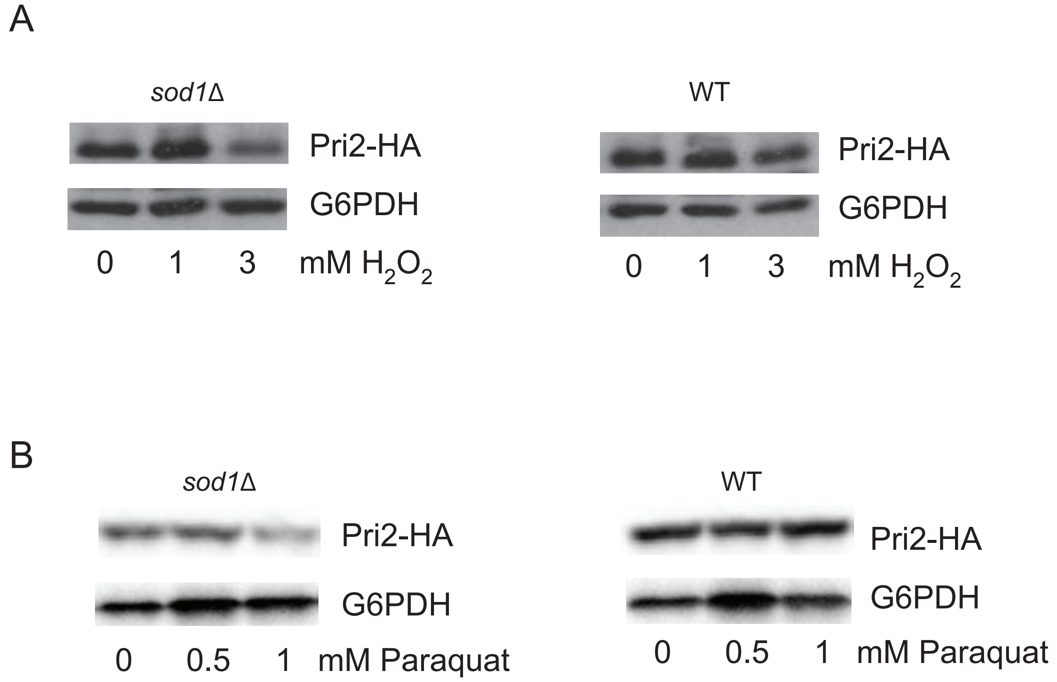

Supplemental Figure S2. Decreased Pri2 protein levels in *sod1Δ* mutant treated with paraquat and H_2O_2

Log-phase culture of sod1Δ and wild-type cells (both containing an integrated PRI2-HA) were treated with **H_2O_2** (A) and paraquat (B) of the indicated concentrations for 3 h at 30°C before being harvested for protein extraction. Protein extracts were resolved by SDS-PAGE and probed for Pri2 and G6PDH as a loading control.
